# Complete Correction of Brain and Spinal Cord Pathology in Metachromatic Leukodystrophy Mice

**DOI:** 10.3389/fnmol.2021.677895

**Published:** 2021-05-21

**Authors:** Emilie Audouard, Valentin Oger, Béatrix Meha, Nathalie Cartier, Caroline Sevin, Françoise Piguet

**Affiliations:** ^1^NeuroGenCell, Institut du Cerveau et de la Moelle Épinière, ICM, Inserm U 1127, CNRS UMR 7225, Sorbonne Université, Paris, France; ^2^Bicêtre Hospital, Neuropediatrics Unit, Le Kremlin Bicêtre, Paris, France

**Keywords:** metachromatic leukodystrophy, aav, gene therapy, intravenous injection (i.v.), sulfatide accumulation, lysosomal storage disease

## Abstract

Metachromatic leukodystrophy (MLD) is a lysosomal storage disorder characterized by accumulation of sulfatides in both glial cells and neurons. MLD results from an inherited deficiency of arylsulfatase A (ARSA) and myelin degeneration in the central and peripheral nervous systems. Currently, no effective treatment is available for the most frequent late infantile (LI) form of MLD after symptom onset. The LI form results in rapid neurological degradation and early death. ARSA enzyme must be rapidly and efficiently delivered to brain and spinal cord oligodendrocytes of patients with LI MLD in order to potentially stop the progression of the disease. We previously showed that brain gene therapy with adeno-associated virus serotype rh10 (AAVrh10) driving the expression of human ARSA cDNA alleviated most long-term disease manifestations in MLD mice but was not sufficient in MLD patient to improve disease progression. Herein, we evaluated the short-term effects of intravenous AAVPHP.eB delivery driving the expression of human ARSA cDNA under the control of the cytomegalovirus/b-actin hybrid (CAG) promoter in 6-month-old MLD mice that already show marked sulfatide accumulation and brain pathology. Within 3 months, a single intravenous injection of AAVPHP.eB-hARSA-HA resulted in correction of brain and spinal cord sulfatide storage, and improvement of astrogliosis and microgliosis in brain and spinal cord of treated animals. These results strongly support to consider the use of AAVPHP.eB-hARSA vector for intravenous gene therapy in symptomatic rapidly progressing forms of MLD.

## Introduction

Metachromatic leukodystrophy (MLD) is a lysosomal storage disorder (LSD) caused by an inherited deficiency of arylsulfatase A (ARSA; EC 3.1.6.8) (Gieselmann and Krägeloh-Mann, 2006). ARSA enzyme catalyzes the first step in the degradation pathway of 3-O-sulfogalactosylceramides (sulfatides). Patients with MLD develop neurological symptoms that result from sulfatide accumulation in oligodendrocytes in the central nervous system (CNS) and Schwann cells in the peripheral nerves (PNS). Sulfatides also accumulate in brain neurons, contributing significantly to additional pathology (Wittke et al., 2004; Gieselmann and Krägeloh-Mann, 2006). Three clinical forms of MLD have been described, based on the age of symptom onset: late infantile, juvenile and adult forms (Gieselmann and Krägeloh-Mann, 2010; Van Rappard et al., 2015). In the case of late-onset forms (first symptoms after 4 years), which are more variable and progress more slowly, allogenic hematopoietic stem cell transplantation (Allo-HSCT) may modify their natural history if performed in pre-symptomatic or early symptomatic patients (Boucher et al., 2015; Groeschel et al., 2016). In the early onset forms (first symptoms before 4 years, the most frequent phenotype of the disease), the disease progresses very rapidly toward severe motor and cognitive regression and premature death, no available therapy being effective once patients are symptomatic. A clinical trial using autologous hematopoietic stem cell transplantation of CD34 cells corrected with lentiviral vector overexpressing human ARSA (HSCT-GT) has shown very encouraging results and provides strong evidence of clinical benefit in pre-symptomatic patients with late infantile MLD (LI-MLD) and pre or early symptomatic patients with early juvenile MLD (EJ-MLD; NCT01560182^1^) (Biffi et al., 2013; Sessa et al., 2016). This treatment will likely become the gold standard treatment for pre-symptomatic MLD patients. However, HSCT-GT failed to stop or slow-down the disease in LI-MLD once patients are symptomatic. Enzyme replacement therapy is another option (Platt and Lachmann, 2009), but the lysosomal enzyme does not cross the blood-brain-barrier (BBB) efficiently, even if results obtained *in vitro* and in MLD mice suggest that ARSA is able to cross the BBB to some extent (Matzner et al., 2009; Matthes et al., 2011). Enzyme replacement therapy using intrathecal delivery of recombinant ARSA enzyme could be an alternative. Results of a phase I-II trial, performed in early symptomatic LI-MLD patients has been published (í Dali et al., 2020), vouching for a good safety profile and some trend to a less pronounced motor function decline over time in patients receiving the highest dose. A phase II-III clinical trial is ongoing (NCT03771898; see text footnote 1) to evaluate the efficacy profile.

However, no effective treatment is currently available for symptomatic patients with early onset forms of MLD. Arresting their rapid neurological degradation by delivering rapidly and efficiently ARSA enzyme into oligodendrocytes, and likely into neurons in brain but also in spinal cord and peripheral nerves, before irreversible damage occurs is crucial.

Previously, we demonstrated that intracerebral gene therapy using an adeno-associated vector of serotype 5 (AAV5) or AAVrh10 to express the human ARSA had a long-term efficacy in MLD mice (Sevin et al., 2006, 2007; Piguet et al., 2012), and more recently the efficacy of these vectors in normal non-human primates (Colle et al., 2009; Zerah et al., 2015). A long-term decrease in sulfatide storage was observed in the brain of treated mice at the neuropathological and biochemical levels, and effect of neuroinflammation was demonstrated in the brain. A Phase I/II clinical trial was initiated in four MLD children (aged between 9 months and 5 years, either pre-symptomatic or early symptomatic), using intracerebral delivery of AAVrh10-hARSA (NCT01801709; see text footnote 1). Despite long-lasting restoration of ARSA activity in the cerebrospinal fluid (CSF, to 20–70% of values in controls), treatment was not able to prevent or even stabilize the disease (Sevin et al., 2018). Lessons of this trial was that intracerebral delivery is probably not enough to achieve sufficient ARSA activity rescue in the whole brain but also in the spinal cord.

Among new serotypes of AAV, AAVPHP.eB was recently described as strongly efficient to cross the BBB after intravenous delivery and lead to an efficient brain and spinal cord transduction in mouse models (Deverman et al., 2016; Chan et al., 2017).

Therefore, as an intravenous delivery of this vector could potentially be more efficient than an AAVrh10 vector, we evaluated, at the neuropathological and biochemical levels, the short-term efficiency of a single intravenous injection of AAVPHP.eB-hARSA-HA vector in 6-month-old MLD mice that have already accumulated marked amounts of sulfatides. Herein, we demonstrate that a single intravenous injection of AAVPHP.eB-hARSA-HA results in robust and diffuse expression of ARSA enzyme in the brain and the spinal cord of MLD treated animals. Importantly, the injection allowed us to correct, within 3 months, the sulfatide accumulation and the neuropathology in brain and spinal cord.

## Materials and Methods

### Adeno-Associated Viral Vector Construction and Production

AAV vectors were produced and purified by Atlantic Gene therapies (Translational Vector Core Research grade services, Nantes, France). AAVPHP.eB-CAG ARSA-HA was produced by cloning the HA tag to the ARSA sequence under the CAG promoter (Piguet et al., 2012). The viral constructs for pAAVPHP.eB-CAG-hARSA-HA contained the expression cassette consisting of the human ARSA genes, driven by a CMV early enhancer/chicken β-actin (CAG) synthetic promoter surrounded by inverted terminal repeats (ITR) sequences of AAV2. Plasmid for AAVPHP.eB was obtained from Addgene (United States). The final titer of the batch was 4.10^12^ vector genomes (vg)/ml.

### Animal Model

All animal studies were performed in accordance with local and national regulations and were reviewed and approved by the relevant institutional animal care and use committee. The experiments were carried out in accordance with the European Community Council directive (2010/63/EU) for the care and use of laboratory animals. Our protocol was approved by the European community council directive (2010/63/EU) (No. 17303). Moreover, the use of GMOs has been validated by the Haut Commissariat aux Biotechnologies (Number 5463).

ARSA-deficient mice (KO ARSA mice) were bred from homozygous founders on a 129/Ola strain (Hess et al., 1996) and heterozygous mice were generated to be control mice. Mice were housed in a pathogen free animal facility in a temperature-controlled room and maintained on a 12-h light/dark cycle. Food and water were available *ad libitum*.

### Injection of AAV Vector

Female and male KO ARSA mice were anesthetized by isoflurane (2% induction). Animals were injected at 6 months of age by intravenous retro-orbital delivery (Yardeni et al., 2011) with saline (NaCl 0.9%) solution or AAVPHP.eB-hARSA-HA (5.10^11^vg total). Retro-orbital administration of the vector was preferred to tail vein injection based on preliminary results that demonstrated a better efficiency to target CNS and decrease liver transduction. Three groups of animals were performed: wild-type (WT, *n* = 3), untreated (NT, *n* = 8) and treated (AAV, *n* = 8) KO ARSA mice. For each group males and females were equally divided so that treatment efficacy is evaluated in both genders. The injected dose was determined according to previous results of dose ranging study on WT animals and evaluation of transduction efficacy.

### Tissue Preparation

Animals were sacrificed by an intraperitoneal administration of a lethal dose of Euthasol (180 mg/kg, Vetcare) 3 months after treatment. Mice were perfused intracardiacally with phosphate buffered saline (PBS). Brain, spinal cord, sciatic nerve, heart, liver, gall gladder, lung, spleen and kidney were collected for analysis. Different structures of a cerebral hemisphere (cortex, striatum, cerebellum, pons and rest of brain) were dissected and frozen in liquid nitrogen. Sciatic nerve, heart, liver, lung, spleen and kidney were directly frozen in liquid nitrogen and stored in −80°C. For DNA and protein extraction from the same samples, tissue samples were crushed in liquid nitrogen and divided into two equals parts. A cerebral hemisphere, a portion of spinal cord, sciatic nerve and gall bladder were post-fixed overnight in 4% paraformaldehyde (PFA)/PBS1X. Samples were rinsed three times in PBS 1X and cryoprotected in 30% sucrose/PBS1X. Tissue are embedded Tissue-Tek OCT compound (VWR International) and cut into 14-μm sagittal section of brain or transversal section of spinal cord or 4 μm longitudinal section of sciatic nerve or transversal section of gall bladder in cryostat (Leica, Langham, TX). Cryosections were dried at room temperature and stored at −20°C.

### Quantitative PCR

DNA was extracted from brain, spinal cord and peripheral organs using chloroform/phenol protocol. AAVPHP.eB-hARSA-HA vector genome copy numbers were measured by quantitative PCR in cortex, striatum, cerebellum, pons, rest of brain, spinal cord and peripheral organs using the Light Cycler 480 SYBR Green I Master (Roche, France) as described (Sevin et al., 2006). The results (vector genome copy number per cell) were expressed as n-fold differences in the transgene sequence copy number relative to the Adck3 gene copy as internal standard (number of viral genome copy for 2N genome). Primers sequence for qPCR were: *human Arsa* (forward 5′-TCA CTG CAG ACA ATG GAC CTG A-3′, reverse 5′-ACC GCC CTC GTA GGT CGT T-3′) and *Adck3* (forward 5′-CCA CCT CTC CTA TGG GCA GA-3′, reverse 5′-CCG GGC CTT TTC AAT GTC T-3′).

### Protein Extraction and ARSA Expression and Activity Quantification

Samples were homogenized in 0.3 ml of lysis buffer (100 mM Trizma base, 150 mM NaCl, 0.3% Triton; pH 7) and incubated for 30 min on ice and centrifuged. The supernatant was collected for the determination of (1) protein content (bicinchoninic acid [BCA] protein assay kit; Pierce Biotechnology/Thermo Fisher Scientific, Rockville, IL); (2) ARSA activity, using the artificial p-nitrocatechol sulfate (pNCS) substrate assay (Sigma-Aldrich, France) (Bass et al., 1970; Piguet et al., 2012). Assays were performed in triplicate and results are expressed as nanomoles of 4-nitrocatechol (4NC) per hour per milligram of protein. And (3) the concentration of recombinant hARSA using an indirect sandwich ELISA specific for human ARSA as described (Matzner et al., 2000), using 2 specific non-commercial antibodies (Kind Gift from Pr. Gielselmann, Bonn). Assays were performed in duplicate and results are expressed as nanograms of hARSA per milligram of protein. All samples were quantified in duplicates.

### Histopathology

To evaluate sulfatide storage, frozen sections were postfixed in 4% PFA, stained with Alcian blue (A5268; Sigma-Aldrich) (0.05% in 0.025 M sodium acetate buffer, pH 5.7, containing 0.3 M MgCl2 and 1% PFA), rinsed in the same buffer without dye, counterstained with fast red (229113; Sigma-Aldrich) and mounted as previously described (Piguet et al., 2012).

Immunohistochemical labeling was performed with the ABC method. Briefly, tissue sections are treated with peroxide (0.9% H_2_O_2_/0.3% Triton/PBS) for 30 min to inhibit endogenous peroxidase. Following washes with PBS, sections are incubated with the blocking solution (10% goat serum in PBS/0.3% TritonX-100) for 1 hr. The primary antibodies [rabbit anti-Calbindin (CB38; Swant, 1:10 000); mouse anti-GFAP (G3893, Sigma-Aldrich; 1:400); rabbit anti-Iba1 (019-19741, Wako; 1:500)] are diluted in blocking solution and incubated on tissue sections overnight at 4°C. After washes in PBS, sections are sequentially incubated with goat anti-rabbit or anti-mouse antibody conjugated to biotin (Vector Laboratories) for 30 min at room temperature, followed by the ABC complex (Vector Laboratories). After washes in PBS, the peroxidase activity is detected with diaminobenzidine as chromogen (Dako, Carpinteria, CA). In some cases, slides are counterstained with hematoxylin. The slides are mounted with Eukitt (VWR International). Slices are acquired at 20X by using a slide scanner (NanoZoomer2.ORS, Hamamatsu).

Tissue cryosections were permeabilized with PBS/0.3%TritonX-100 for 15 min and saturated with PBS/0.3% Triton/10% horse serum (HS) for 45 min. The primary antibodies were diluted in the saturation solution and incubated 1 h at 37°C. After washes in PBS/0.1% Triton, the secondary antibodies and DAPI were diluted in PBS/0.1% Triton/10% HS and added for 1 h at room temperature. After washes in PBS/0.1% Triton, the slides were mounted with fluorescent mounting medium (F4680; Sigma-Aldrich). Primary antibodies for immunofluorescence were rabbit polyclonal anti-hARSA (kind gift from V. Gieselmann and U. Matzner, Bonn, Germany; 1:1,000) and mouse anti-Lamp1 (1D4B, DSHB, 1:200). Secondary antibodies were diluted 1:1,000 and were donkey anti-rabbit/AlexaFluor 594 and anti-mouse/AlexaFluor 488. Pictures were taken with a Confocal SP8 Leica DLS Inverted (Leica). For all images, brightness and contrast were adjusted with Image J software after acquisition to match with the observation. All histological studies were assessed blinded by two investigators.

### Stereological Cell Counts

Stereological counts were performed by two independent investigators, blind for both genotypes and treatments, using Image J software. All quantifications were done on three sections of brain and of spinal cord for each animal (*n* = 3 for WT, *n* = 8 for NT, and *n* = 3 for AAV). For Alcian staining, the number of sulfatide storage inclusions were quantified in three random areas of the cortex, the corpus callosum and the fimbria. For GFAP and Iba1 labeling, the number of positive cells were evaluated in three random areas of the cortex and corpus callosum or the center of cerebellum white matter.

In the spinal cord, for sulfatide storage, GFAP and Iba1, a hemi-section was counted. All results were assessed per mm^2^ and expressed as the mean ± SEM.

### Statistical Analysis

Data were analyzed using GraphPad Prism 8 software. The statistical significance of values among groups was evaluated by ANOVA, followed by the least significant difference *t*-test. All values used in figures and text are expressed as mean ± standard error of the mean (SEM). Differences were considered significant at *p* < 0.05.

For all graphs, a special symbol has been assigned for each individuals so that it is possible to correlate between ARSA expression and astrogliosis, sulfatide accumulation and so.

## Results

### Validation of pAAV-CAG-hARSA-HA Plasmid *in vitro*

After hARSA-HA cloning in the pAAV plasmid and validation by sequencing, an *in vitro* assay based on 293T cells transfection with pAAV-CAG-hARSA-HA plasmid was performed. We demonstrated hARSA-HA expression using HA staining, in transfected cells as well as a significant increase in ARSA activity in supernatant of transfected cells, up to 90-folds compared to non-transfected cells, 72 h after transfection (Supplementary Data and Supplementary Figure 1). These data confirmed the functionality of the pAAV-CAG-hARSA-HA plasmid. The AAVPHP.eB-hARSA-HA vector was produced as described.

### Widespread Distribution and Expression of AAVPHP.eB-hARSA-HA in the CNS of KO ARSA Mice

**FIGURE 1:**
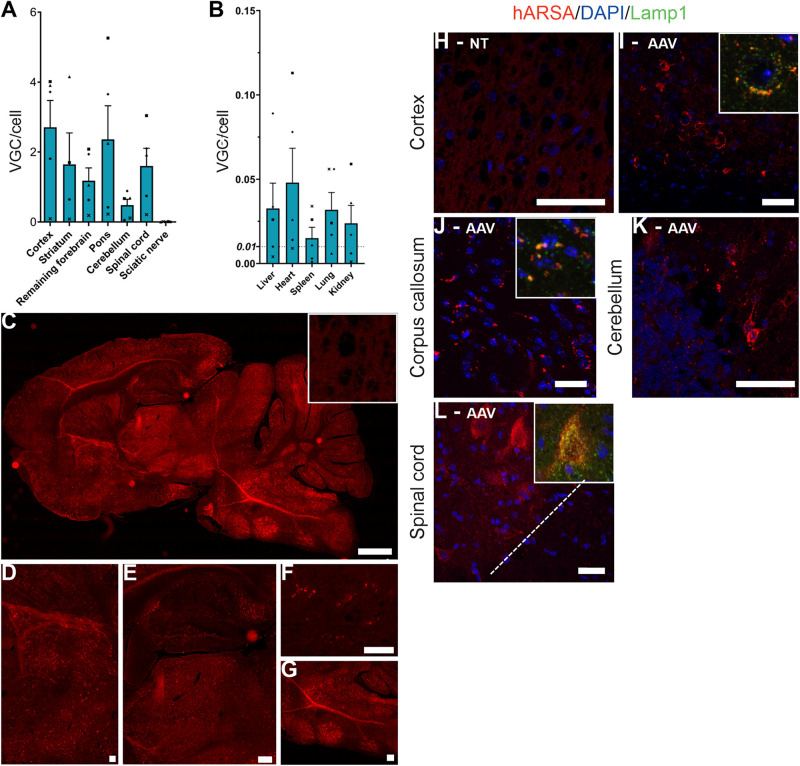
AAVPHP.eB-hARSA-HA efficiently transduce central nervous system. **(A,B)** Biodistribution of the AAVPHP.eB-hARSA-HA in central nervous system **(A)** and peripheral organs **(B)** in 9-month-old KO ARSA mice, 3 months after intravenous injection of AAVPHP.eB-hARSA-HA (*n* = 5). VGC for vector genome copy number per 2n genome. **(C–G)** Immunofluorescence detection of hARSA (red) on sagittal sections in the brain **(C)** and high magnification of different brain areas **(D–G)** i.e., Striatum **(D)**, hippocampus and thalamus **(E)**, corpus callosum **(F)** and cerebellum **(G)** in 9-month-treated KO ARSA mice with AAVPHP.eB-hARSA-HA. Inset is a sagittal section of cortex in control mice. **(H–L)** Immunofluorescence detection of hARSA (red) and of Lamp1 (green) on sagittal sections in the cortex **(H,I)**, corpus callosum **(J)** and cerebellum **(K)** and on coronal sections of spinal cord **(L)** in 9-month-old untreated **(H)** or treated **(I–L)** KO ARSA mice with AAVPHP.eB-hARSA-HA. Nuclei are stained in blue. Insert in (**I**,**J**,**L**) shows a co-localization of hARSA in lysosome (Lamp1, green). Data are represented as mean ± SEM. Scale Bars: 1,000 μm **(C)**; 200 μm **(E,G)**; 100 μm **(E)** and 50 μm **(F,H–L)**.

Treated KO ARSA mice (*n* = 8) mice received a single intravenous injection of AAVPHP.eB-hARSA-HA. Treatment was well tolerated, no adverse event was observed in mice injected with the AAVPHP.eB vector, attesting for the safety of the procedure. Vector injection resulted in a widespread transduction of CNS, with a mean of 2.71 ± 0.76 vector genome copies per cell (VGC) in the cortex, 1.6 VGC ± 0.90 in the striatum, 2.3 VGC ± 0.96 in the pons, 1.2 VGC ± 0.37 in the remaining forebrain, 0.5 VGC ± 0.17 in the cerebellum and 1.6 VGC ± 0.50 in the spinal cord (Figure 1A) in treated mice. The mean number of VGC in peripheral organs was less than 0.05 VGC, indicating a low peripheral transduction of the vector (Figure 1B).

In accordance with the biodistribution profile, hARSA expression was detected by immunofluorescence studies in several areas of brain (Figure 1C) of treated KO ARSA mice, such as striatum (Figure 1D), hippocampus, thalamus (Figure 1E), corpus callosum (Figures 1F,J), pons and cerebellum (Figures 1G,K). Moreover, hARSA-positive cells were also detected in the spinal cord (Figure 1L) of treated mice. As a negative control, hARSA protein expression was not detected in untreated KO ARSA mice (Figure 1H). To be active, ARSA enzyme needs to be targeted to the lysosome. Proper lysosomal localization was confirmed by co-staining with anti-hARSA and anti-Lamp1 (lysosomal marker) antibodies, performed on brain and spinal cord sections of treated KO ARSA mice. A colocalization of hARSA and Lamp1 was observed in different areas of CNS (Inset, Figures 1I–K), indicating hARSA is correctly localized in lysosomes and thus could catabolize sulfatides.

### hARSA Activity and Expression in CNS of Treated KO-ARSA Mice

**FIGURE 2:**
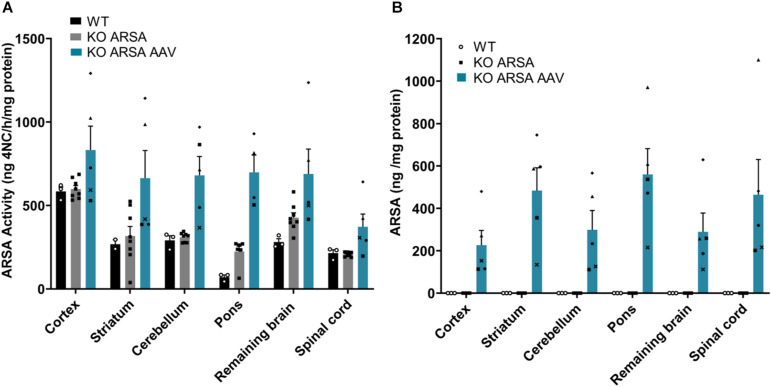
**(A)** ARSA activity in several brain regions and spinal cord in 9-month-old wild-type (*n* = 3), untreated (*n* = 7) and treated KO ARSA (*n* = 5) mice with AAVPHP.eB-hARSA-HA. **(B)** Arylsulfatase A (ARSA) expression (ng/mg protein) assessed by ELISA in several brain regions and spinal cord in 9-month-old wild-type (*n* = 3), untreated (*n* = 3) and treated KO ARSA (*n* = 5) mice with AAVPHP.eB-hARSA-HA. Data are represented as mean ± SEM.

To validate the functionality of recombinant hARSA in treated KO ARSA mice, ARSA activity was measured in different structures of the CNS. We demonstrated a clear trend to ARSA over activity in the cortex, pons, cerebellum and spinal cord in treated KO ARSA mice (Figure 2A). Moreover, expression of recombinant hARSA assessed by ELISA in several structures of brain and the spinal cord with a mean to 326 ng ARSA/mg protein in treated KO mice whereas it was not detected in WT and untreated mice (Figure 2B). We demonstrated a high hARSA expression in the brain and the spinal cord in treated KO ARSA. To conclude, treated mice with AAVPHP.eB-hARSA-HA express high levels of functional ARSA enzyme.

### Significant Improvement of MLD Pathophysiology in Treated KO ARSA Mice With AAVPHP.eB-ARSA-HA

#### Complete Correction of Sulfatide Storage in CNS of Treated KO ARSA

**FIGURE 3:**
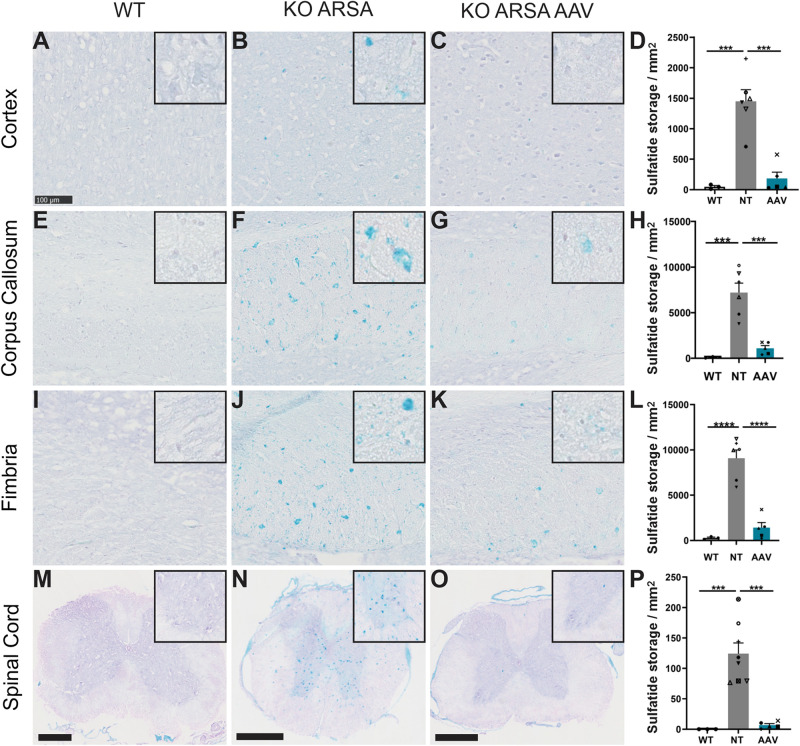
Correction of sulfatide storage in brain and spinal cord of treated KO-ARSA mice, 3 months after treatment **(A–C,E–G,I–K,M–O)**. Alcian blue staining in cortex **(A–C)**, corpus callosum **(E–G)**, fimbria **(I–K)** and spinal cord **(M–O)** of wild-type (WT; **A,E,I,M**), untreated (KO ARSA; **B,F,J,N**) and AAVPHP.eB-hARSA-HA treated (KO ARSA AAV; **C,G,K,O**) KO ARSA mice. Inserts are high magnification of tissue section to show the absence or the presence of sulfatide storage. **(D,H,L,P)** Quantification of sulfatide storage per mm^2^ in cortex **(D)**, corpus callosum **(H)**, fimbria **(L)** and spinal cord **(P)** of WT (*n* = 3), untreated (NT, *n* = 6–8) and treated (AAV, *n* = 5) KO ARSA mice. Data are represented as mean ± SEM. Scale bars: 100 μm expected for **(M–O)**, scale bars: 500 μm. ***p < 0.001; ****p < 0.0001.

Sulfatide accumulation starts during fetal development, is obviously detectable at 3 months of age in the CNS of KO ARSA mice (corpus callosum and pons) and then increases progressively with age (personal non-published data). To assess the efficiency of intravenous administration of AAVPHP.eB-ARSA-HA vector to decrease sulfatide storage, alcian staining was performed in the brain, spinal cord, sciatic nerve and gall bladder sections of untreated and treated KO ARSA animals and compared to wild-type control. Nine-month-old untreated KO ARSA mice display massive sulfatide storage in brain, spinal cord, sciatic nerve and gall bladder compared to WT mice (Figure 3 and Supplementary Figure 2). In 9-month-old treated animals, 3 months after intravenous injection, AAVPHP.eB-ARSA-HA vector had significantly decreased the sulfatide storage in brain and spinal cord of treated KO ARSA mice (Figures 3C,G,K,O). This was confirmed by the quantification of the number of sulfatide storage inclusions in the cortex, corpus callosum, fimbria and spinal cord (Figures 3D,H,L,P). Indeed, a tremendous decrease of sulfatide accumulation was observed in treated mice that were almost similar to WT mice for brain and spinal cord. However, sulfatide storage was not improve in gall bladder and sciatic nerve of treated animals that remained similar to untreated animals (Supplementary Figure 2).

#### No Abnormalities of Purkinje Cells in KO ARSA Mice

In this mouse model, the abnormalities of Purkinje cells are detected around 12 months (Hess et al., 1996). To confirm the absence of abnormalities in Purkinje cells of 9-month-old mice, an immunochemical staining was performed with anti-calbindin antibody on the brain sections of different groups. No abnormalities in the number of Purkinje cells were observed in 9-month-old KO ARSA mice (data not shown). This result confirms the absence of phenotype in cerebellum of 9-month-old KO ARSA mice.

#### AAVPHP.eB-hARSA-HA Treatment Rescue Neuroinflammation in MLD Mouse Model

**FIGURE 4:**
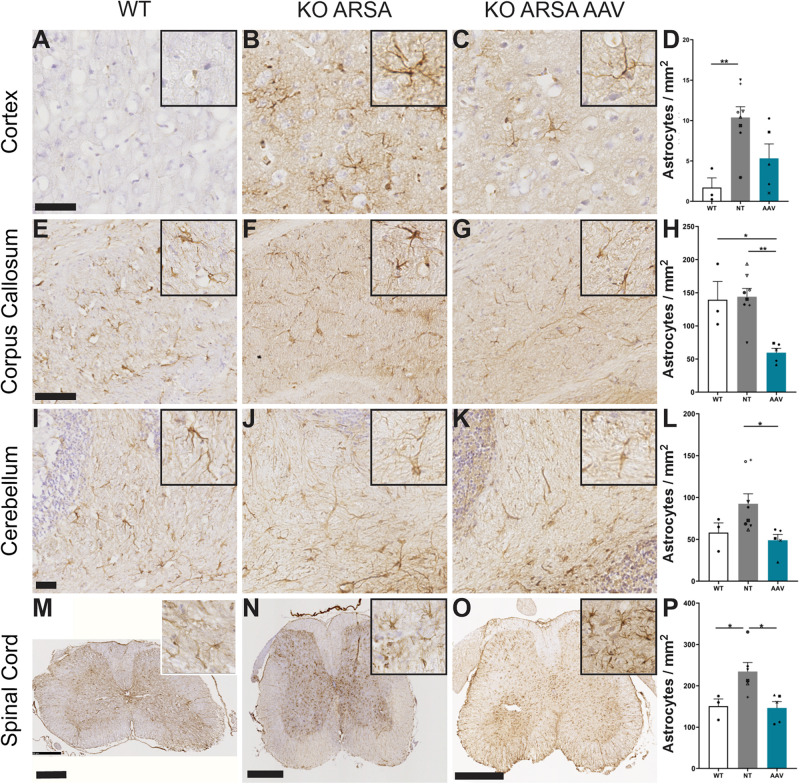
Correction of astrogliosis in brain and spinal cord of treated KO-ARSA mice, 3 months after treatment. **(A–C,E–G,I–K,M–O)** Immunohistochemistry of GFAP in cortex **(A–C)**, corpus callosum **(E–G)**, cerebellum **(I–K)** and spinal cord **(M–O)** of wild-type (WT; **A,E,I,M**), untreated (KO ARSA; **B,F,J,N**) and AAVPHP.eB-hARSA-HA treated (KO ARSA AAV; **C,G,K,O**) KO ARSA mice. Insets are high magnification of tissue section. **(D,H,L,P)** Quantification of GFAP-positive cells per mm^2^ in cortex **(D)**, corpus callosum **(H)**, cerebellum **(L)** and spinal cord **(P)** of WT (*n* = 3), untreated (NT, *n* = 6–8) and treated (AAV, *n* = 5) KO ARSA mice. Data are represented as mean ± SEM. Scale bars: 50 μm **(A–C)**, 100 μm **(E–H)**, 500 μm **(M–O)**. *p < 0.05; **p < 0.0.

Astrogliosis and microgliosis are two hallmarks of MLD pathology that are present in MLD mouse model (Sevin et al., 2006, 2007; Piguet et al., 2012). To assess the effect of AAVPHP.eB-hARSA-HA treatment on astrogliosis and microgliosis in KO ARSA mice, an immunohistochemical staining was performed with anti-GFAP or anti-Iba1 antibodies on the brain and spinal cord sections of different groups of animals. A significant increase of GFAP-positive cells was observed in cortex (Figures 4A–D) and spinal cord (Figures 4M–P) of 9-month-old untreated mice compared to WT mice. In the cerebellum, an increase of GFAP-positive cells was also detected in KO ARSA mice, compared to WT animals, even if not significant (Figures 4I–L). No astrogliosis was detected in the corpus callosum of untreated mice (Figures 4E–H). Three months after injection with AAVPHP.eB-hARSA-HA, a significant decrease of astrogliosis was observed in the spinal cord of treated KO ARSA mice, as well as a trend to improvement in the cortex and cerebellum, vouching for a clear therapeutic effect (Figure 4).

**FIGURE 5:**
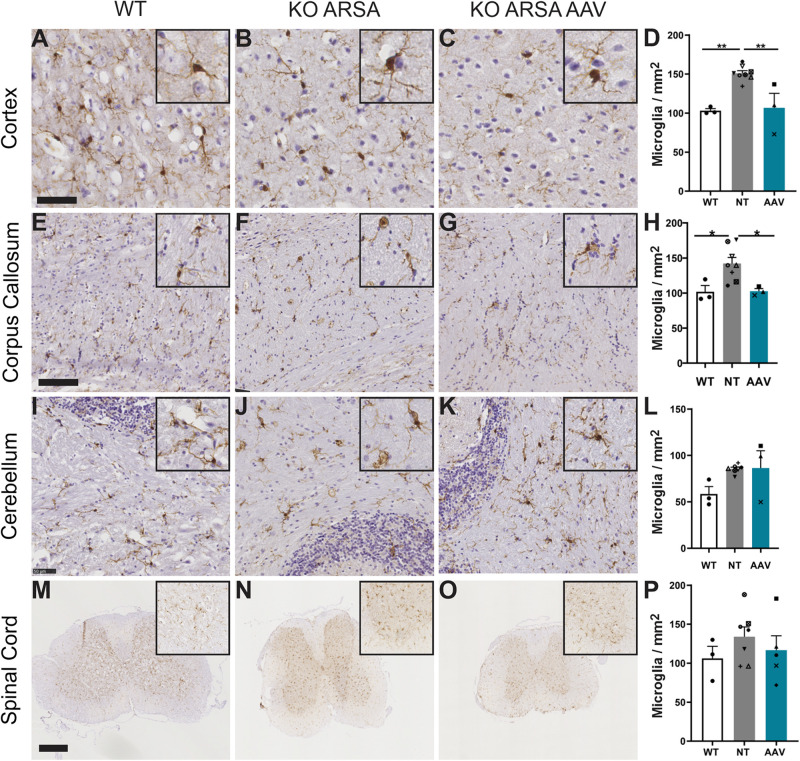
Correction of microgliosis in cortex and corpus callosum of treated KO-ARSA mice, 3 months after treatment. **(A–C,E–G,I–K,M–O)** Immunohistochemistry of Iba1 in cortex **(A–C)**, corpus callosum **(E–G)**, cerebellum **(I–K)** and spinal cord **(M–O)** of wild-type (WT; **A,E,I,M**), untreated (KO ARSA; **B,F,J,N**) and AAVPHP.eB-hARSA-HA treated (KO ARSA AAV; **C,G,K,O**) KO ARSA mice. Insets are high magnification of tissue section. **(D,H,L,P)** Quantification of Iba1-positive cells per mm^2^ in cortex **(D)**, corpus callosum **(H)**, cerebellum **(L)** and spinal cord **(P)** of WT (*n* = 3), untreated (NT, *n* = 6–8) and treated (AAV, *n* = 5) KO ARSA mice. Data are represented as mean ± SEM. Scale bars: 50 μm **(A–C,I–K)**, 100 μm **(E–H)**, 500 μm **(M–O)**.*p < 0.05; **p < 0.01.

A significant increase of Iba1-positive cells was observed in cortex and corpus callosum of untreated KO ARSA mice, compared to WT mice (Figures 5A–H). This was not observed in the cerebellum and in spinal cord of untreated mice compared to WT mice (Figures 5M–P), even if microglia had tendency to increase in both these structures (Figures 5I–L). Three months after AAVPHP.eB-hARSA-HA injection, a significant decrease in microgliosis was observed in both cortex and corpus callosum of treated mice (Figures 5A–H), indicating a positive therapeutic effect. No difference of microgliosis was shown in cerebellum and spinal cord of treated mice compared to two other groups.

In summary, makers of neuroinflammation observed in the CNS of 9-month-old KO ARSA mice was significantly reduced after intravenous administration of the AAVPHP.eB-hARSA-HA therapeutic vector.

## Discussion

Treating early onset forms of MLD is a huge challenge, given the rapidity of the neurologic deterioration in these forms of the disease. Given the rapid and devastating progression of cerebral disease in patients, the therapeutic challenge is to deliver rapidly and efficiently ARSA enzyme or gene in both neurons and oligodendrocytes and not only in the brain but also in the spinal cord. This is notably the case for patients already symptomatic in which HSCT or HSCT-GT are insufficient (Sessa et al., 2016).

Very promising results let us consider that HSCT-GT may become in the future the standard of care for patients with early onset MLD at a pre-symptomatic stage, justifying an expansion of the newborn screening for MLD. However, its effect is dependent on a delayed onset of action (12–18 months), which is a problem for these very rapidly progressing diseases. Thus, up to now, no therapy is available once symptoms are already present, which is the main clinical situation in the daily life for pediatricians. Intrathecal ERT, currently under clinical evaluation, could be helpful and act very quickly but would require lifelong repeated injections.

*In vivo* gene therapy is attracting growing interest for the treatment of neurodegenerative diseases, including MLD, with the goal to act quickly (few weeks) and achieve, after a “one-shot” procedure, long-lasting expression of the therapeutic gene. In our intracerebral AAVrh.10 gene therapy trial (NCT01801709), despite long-lasting restoration of ARSA activity in the CSF, we failed to demonstrate any clinical effect, even in pre-symptomatic patients with LI-MLD (Sevin et al., 2018). Inconstant results were observed in clinical trials using intracerebral GT for other lysosomal diseases (MPSIIIA, MPSIIIB, LINCL) (Tardieu et al., 2014, 2017; Sondhi et al., 2020). New routes of administration (intra-CSF, intravenous) are currently under clinical evaluation, most of them using AAV9 vector. Interestingly, a combined approach, using both intravenous gene therapy (with an AAVrh.10 vector) and allogenic HSCT is currently in clinical trial in pre-symptomatic children with infantile Krabbe disease (NCT04693598).

Here, we proposed an intravenous gene therapy approach to rapidly and efficiently deliver ARSA expression both in brain and in spinal cord, using intravenous administration of AAVPHP.eB-hARSA-HA in MLD mice. AAVPHP.eB was recently described as strongly efficient to cross the BBB after intravenous delivery and lead to an efficient brain and spinal cord transduction in mouse models (Deverman et al., 2016; Chan et al., 2017). Moreover, the ARSA KO mouse strain is 129/Ola, which is known to express the LY6A receptor (Hordeaux et al., 2018; Mathiesen et al., 2020), and thus ability of AAVPHP.eB to transduce the BBB.

Our results demonstrate clearly that intravenous AAVPHP.eB-hARSA-HA delivery in MLD mice lead to a broad transduction of brain and spinal cord (Figure 1) without major transduction in peripheral organs (Figure 1), confirming the CNS tropism described by Deverman et al. (2016). No behavioral tests were done in this study due to the age of the mice at the necropsy as rotarod alterations are known to be clear around 12–18 months of age (Sevin et al., 2006, 2007).

Moreover, compare to our previous studies with AAVrh10, mean levels in the brain (striatum, cortex and rest of the brain) is similar between intravenous delivery of AAVPHP.eB-hARSA (330 ng ARSA/mg of protein) than with AAVrh10 intracerebral delivery (325 ng/mg of proteins). However, in the cerebellum, brainstem and spinal cord where no ARSA expression was detected with AAVrh10 (Piguet et al., 2012), intravenous delivery of AAVPHP.eB-hARSA lead to high expression of the hARSA (around 300 ng/mg of protein in the cerebellum; 560 ng/mg of protein in the brainstem and 460 ng/mg of protein in the spinal cord). This strongly demonstrates the efficacy of the intravenous delivery of the AAVPHP.eB to achieve a broad and strong expression of ARSA, which is suitable for therapeutic development.

Intravenous delivery of AAVPHP.eB-hARSA-HA in MLD mice leads to a rapid and complete correction of brain sulfatide storage, one of the hallmarks of the disease (Gieselmann and Krägeloh-Mann, 2006), as our previous approach with intracerebral delivery of AAVrh10-ARSA (Piguet et al., 2012). However, we can demonstrate here a complete correction of sulfatide storage in spinal cord of treated animals. This has never been observed previously with both our AAV5 and AAVrh10 intracerebral approach (Sevin et al., 2006, 2007; Piguet et al., 2012) and even never reported in MLD mice after enzyme replacement therapy (Matzner et al., 2009; Matthes et al., 2012). In addition, the treatment was administrated in 6-month-old mice, an age where the sulfatide storage is already clearly detectable (Hess et al., 1996) and its effectiveness was evaluated only 3 months after injection for which the complete rescue was observed in the CNS. This indicates us that a strong expression of ARSA leads to a rapid reversal of sulfatide storage, which is a crucial advantage for rapidly progressive form of MLD.

The second hallmark of the disease on which we focused was the astrogliosis and microgliosis. Neuroinflammation is a hallmark of many LSDs with CNS involvement, including MLD and has emerged as a key factor in promoting neurodegeneration in these diseases. Astrogliosis and microglia activation are observed in the brain of MLD patients and mice, associated with increased inflammatory cytokines (Jeon et al., 2008; Stein et al., 2015; Thibert et al., 2016). The most commonly accepted hypothesis is that neuroinflammation is due to secondary activation of microglia following phagocytosis of myelin debris containing undegraded material. However, microglia activation and elevation of cytokines have been shown to precede demyelination in MLD mice, suggesting that neuroinflammation may also be a primitive phenomenon (Stein et al., 2015). In our study, we demonstrated a significant improvement of astrogliosis and microgliosis in AAVPHP.eB-hARSA-HA treated mice compared to untreated MLD mice, as efficiently as our previous approach with intracerebral delivery of AAVrh10-ARSA ARSA (Piguet et al., 2012). However, we can demonstrate, in addition, a clear reduction/normalization of neuroinflammation in spinal cord which was never observed previously with our AAV5 and AAVrh10 intracerebral approach (Sevin et al., 2006, 2007; Piguet et al., 2012) neither with the enzyme replacement therapy (Matzner et al., 2009; Matthes et al., 2012).

Altogether, the intravenous injection of AAVPHP.eB-hARSA-HA vector resulted in an unprecedented level of sulfatide and neuropathology corrections, not only in the brain but also in the spinal cord. Those results provide strong support for implementing intravenous AAVPHP.eB gene therapy in MLD patients with rapidly progressive forms of the disease after disease onset, but also in other rapidly progressing leukodystrophies like Krabbe disease. Tolerance and efficacy studies are currently in progress in non-human primates before translation to human patients, in particular to evaluate the dose of AAVPHP.eB that would be required for therapeutical benefit. Preliminary results demonstrated a capacity of the AAVPHP.eB to cross brain barrier and transduce CNS in NHP without sign of toxicity. In NHP, there is no direct homolog of LY6A receptor, however, other key factor could share properties and allow AAVPHP.eB passage across BBB (Huang et al., 2019). In the optic of a clinical application, the existence of preexisting antibodies is a remaining question as AAV is highly prevalent in humans (Fu et al., 2017), that would anyway need a serology prior to injection. This has not been evaluated in mouse has there is no preexisting immunity.

## Data Availability Statement

The original contributions presented in the study are included in the article/Supplementary Material, further inquiries can be directed to the corresponding author/s.

## Ethics Statement

The animal study was reviewed and approved by CE17 Darwin. Written informed consent was obtained from the owners for the participation of their animals in this study.

## Author Contributions

EA and FP designed the experiments. EA, FP, VO, and BM performed the experiments. EA, FP, and CS wrote the manuscript. All authors contributed to the article and approved the submitted version.

## Conflict of Interest

The authors declare that the research was conducted in the absence of any commercial or financial relationships that could be construed as a potential conflict of interest.

## Footnotes

1 http://clinicaltrials.gov
