# Supplementary figures and images for: Complete Correction of Brain and Spinal Cord Pathology in Metachromatic Leukodystrophy Mice
Source: Front Mol Neurosci. 2021 May 21;14:677895. doi: 10.3389/fnmol.2021.677895 (PMC8175802; doi:10.3389/fnmol.2021.677895)

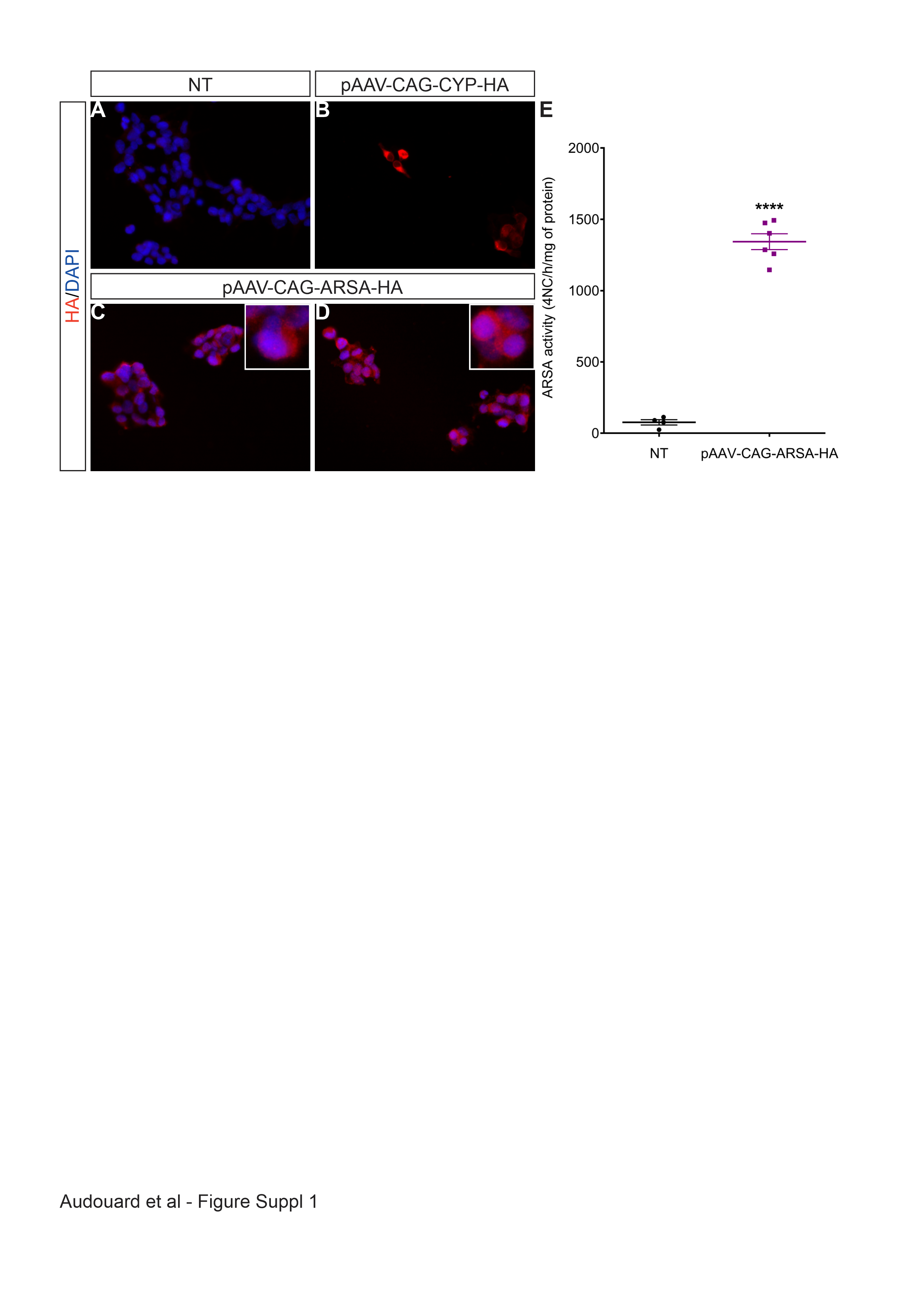

Supplement: Supplementary file 1 [file Image_1.tiff]

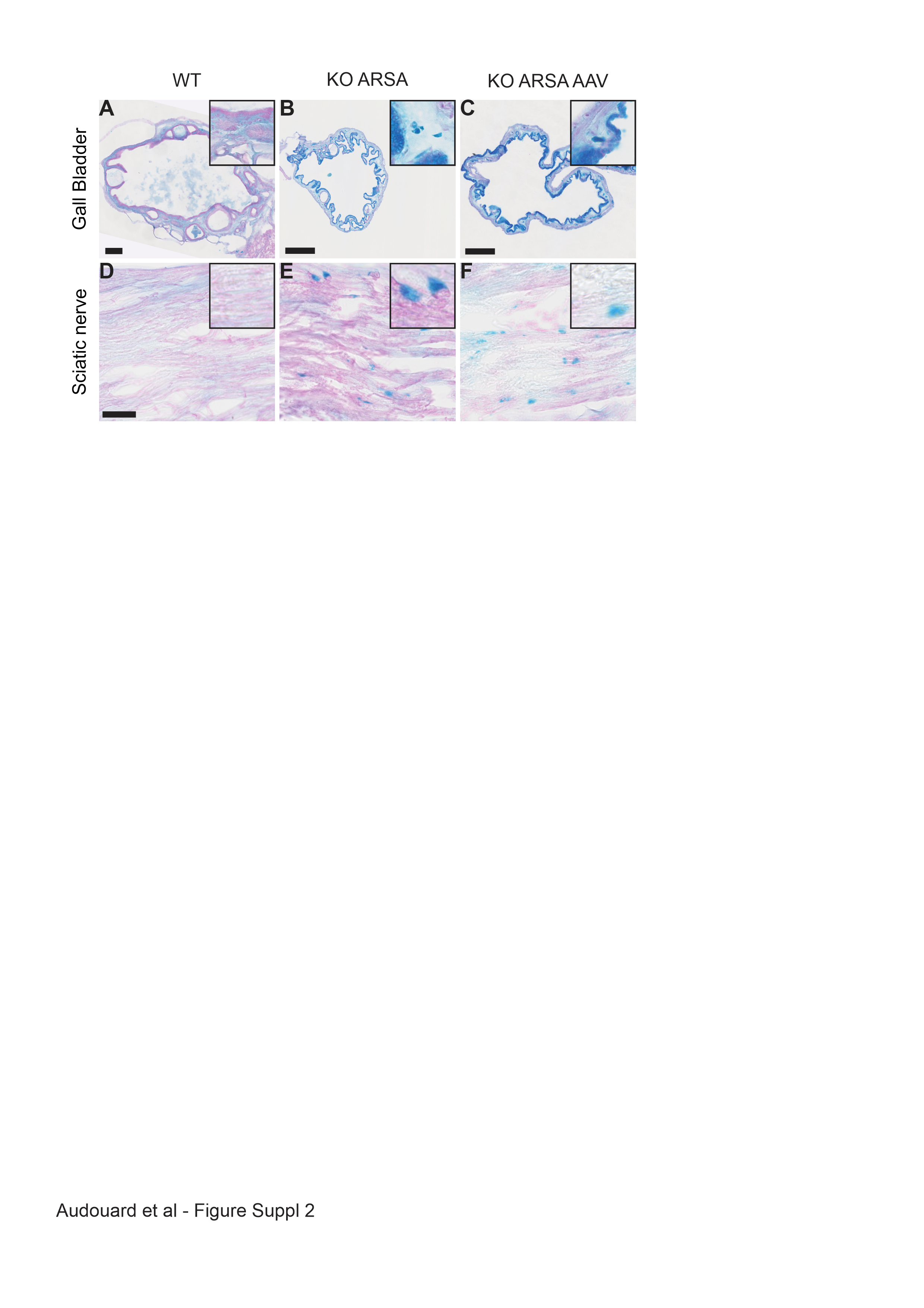

Supplement: Supplementary file 2 [file Image_2.tiff]
