# Supplementary material for: Complete Correction of Brain and Spinal Cord Pathology in Metachromatic Leukodystrophy Mice
Source: Front Mol Neurosci. 2021 May 21;14:677895. doi: 10.3389/fnmol.2021.677895 (PMC8175802; doi:10.3389/fnmol.2021.677895)
Supplement: Supplementary file 3 [file Table_1.DOCX]

Supplementary Material

# Supplementary Data

## In vitro validation of ARSA plasmid

293T cells were transfected with 4µg of plasmid (pAAV-CAG-hARSA-HA or hCYP-HA or without) and lipofectamine (#11668-019, Invitrogen) for 6 hours. Three days after transfection, the supernatants were collected to measure ARSA activity. An immunofluorescence was performed on the 293T cells. Cells were fixed with PFA 4% for 15 min at room temperature. After washes with PBS1X, cells were permeabilized with Triton0.3%/ PBS1X for 15 min. The primary antibody (mouse anti-HA; Biolegend # 901514; 1:500) was incubated with Triton0.1%/NGS5%/PBS1X at 37°C for 1 hour. After washer with triton0.1%/PBS1X, the secondary antibody (goat anti-mouse alexa 488, 1:1000) and dapi were incubated for 1 hour at room temperature. Slides were mounted with fluoromount (F4680; Sigma).

# Supplementary Figures and Tables

## Supplementary Figures

**Supplementary Figure 1:** In vitro validation of the plasmid pAAV-CAG-hARSA-HA by transfection of 293T cells. (A-D) HA staining in non-transfected (NT;A) or transfected cells with control plasmid (CYP-HA; B) or hARSA-HA plasmid (C-D). Nuclei are stained in blue. Analysis have been performed 72h after cell transfection. Insets are high magnification of some cells. (E) ARSA activity quantification on cells lysates form non-transfected and transfected cells. Data are represented as mean +/- SEM. ****p<0.0001.

**Supplementary Figure 2:** Alcian blue staining in gall bladder (A-C) and sciatic nerve (D-F) of wild-type (WT; A,D), untreated (KO ARSA; B, E) and AAVPHP.eB-hARSA-HA treated (KO ARSA AAV; C,F) KO ARSA mice. Insets are high magnification of tissue section to show the presence of sulfatide storage. Scale bars: 100 µm (A-C) and 50µm (D-F).
